# Cytokine gene expression profiling identifies distinct patterns in severe sepsis

**DOI:** 10.1186/cc11795

**Published:** 2012-11-14

**Authors:** J Sarveswaran, NM Orsi, M Cummings, S Homer-Vanniasinkam, D Burke

**Affiliations:** 1 Leeds Teaching Hospitals, Leeds, UK

## Background

In patients with severe sepsis, previous work has suggested that cytokine gene expression profiles may have diagnostic/prognostic potential as biomarkers. Cytokines operate within complex networks; their predictive value may be improved by the simultaneous measurement of multiple cytokines. Cytokines are key inflammatory proteins involved in sepsis. Gene expression profiling has been shown to have a clinical role in the diagnosis of haematological malignancies. Gene expression profiling may allow early, aggressive management in patients predicted to fare badly. This study examined cytokine gene expression profiles in patients with severe sepsis.

## Methods

Patients admitted to the ICU within 24 hours of onset of severe sepsis were recruited. Healthy controls were also recruited. Exclusion criteria included patients on immunosuppressants or chemotherapy and those with a moribund status. Ethical approval (REC W06/Q1107/42) was obtained. Whole blood (1 ml) was taken from the patients and Quantigene RNA extraction techniques were used. The following cytokines were measured in duplicate using the Quantigene plex assay: IL-6, IL-8, IL-10, IL-12p40, IL-12p35, IL-13, IL-17, MCP-1 and MIF. The assay uses branched DNA signal amplification to enable the detection and quantification of multiple RNA targets simultaneously. It has been compared with other techniques favourably [1]. Cytokine mRNA expression was standardised to housekeeper genes succinate dehydrogenase (SDHA) and hypoxanthine-guanine phosphoribosyltransferase (HPRT).

## Results

Sixty-five patients with severe sepsis were included, of whom 27 died within 28 days. Seventeen control samples were included in the study. The groups were age and sex matched. Three cytokines showed marked upregulation in severe sepsis compared with controls; IL-10 (15-fold), IL-18 (15-fold), IL-15 (twofold). However, IL-6, IL-8, IL-12p40, IL-12p35, IL-13, IL-17, MCP-1 all showed a greater than twofold downregulation in severe sepsis. There was no significant difference in profiles between survivors and nonsurvivors of severe sepsis.

## Conclusion

These results show a distinct cytokine gene expression profile in severe sepsis. This includes the upregulation or downregulation of both proinflammatory and anti-inflammatory cytokines. Cytokine gene expression profiling using the Quantigene plex assay is able to demonstrate distinct profiles in patients with severe sepsis. This has the potential to be developed into a diagnostic/prognostic tool with larger studies.
